# Biogeographic patterns of abundant and rare bacterioplankton in three subtropical bays resulting from selective and neutral processes

**DOI:** 10.1038/s41396-018-0153-6

**Published:** 2018-06-07

**Authors:** Yuanyuan Mo, Wenjing Zhang, Jun Yang, Yuanshao Lin, Zheng Yu, Senjie Lin

**Affiliations:** 1 State Key Laboratory of Marine Environmental Science, Marine Biodiversity and Global Change Research Center, College of Ocean and Earth Sciences, Xiamen University, Xiamen, China; 2 Aquatic EcoHealth Group, Key Laboratory of Urban Environment and Health, Institute of Urban Environment, Chinese Academy of Sciences, Xiamen, China; 3 Department of Chemical Engineering, University of Washington, Seattle, WA USA; 4 Department of Marine Sciences, University of Connecticut, Groton, CT USA

## Abstract

Unraveling the relative importance of ecological processes regulating microbial community structure is a central goal in microbial ecology. Here, we used high-throughput sequencing to examine the relative contribution of selective and neutral processes in the assembly of abundant and rare subcommunities from three subtropical bays of China. We found that abundant and rare bacterial taxa were distinctly different in diversity, despite the similar biogeographic patterns and strong distance-decay relationships, but the dispersal of rare bacterial taxa was more limited than that of abundant taxa. Furthermore, the environmental (selective processes) and spatial (neutral processes) factors seemed to govern the assembly and biogeography of abundant and rare bacterial subcommunities, although both factors explained only a small fraction of variation within the rare subcommunity. More importantly, variation partitioning (based on adjusted *R*^2^ in redundancy analysis) showed that spatial factors exhibited a slightly greater influence on both abundant and rare subcommunities compared to environmental selection; however, the abundant subcommunity had a much stronger response to spatial factors (17.3% of pure variance was explained) than that shown by the rare bacteria (3.5%). These results demonstrate that environmental selection and neutral processes explained the similar biogeographic patterns of abundant and rare subcommunities, but a large proportion of unexplained variation in the rare taxa (91.1%) implies that more complex assembly mechanisms may exist to shape the rare bacterial assemblages in the three subtropical bays.

## Introduction

Microbial communities are highly diverse and are crucial drivers of global biogeochemical cycles across ecosystems [1–3]. One common phenomenon is that there are a small number of very abundant taxa, whereas a large number of taxa (the “rare biosphere”) have lower abundance and extremely high diversity [4, 5]; the latter subcommunity is still largely unexplored [6, 7]. The most abundant taxa are thought to be the most important in fluxes of dissolved organic matter and carbon cycling [6, 8]. However, recent evidence has indicated that rare taxa are also key for primary ecosystem functions [9]. For example, many rare taxa contribute to nutrient cycling [10, 11], and they act as a reservoir that can quickly respond to environmental changes to promote community stability in a wide variety of ecosystems [12]. Thus, the study of both abundant and rare microbial diversity and spatial patterns is important. However, it still remains unclear whether rare bacterial taxa have similar biogeography as the abundant taxa in typical subtropical marine ecosystems.

High-throughput sequencing of unparalleled depth coverage permits the accurate examination of biogeographic dynamics of marine microbiota [13–16], whereas the mechanisms structuring microbial communities are not well understood. There is growing evidence for the importance of two different processes acting to determine the composition of microbial communities. One is niche-based (environmental selection-related) process [17, 18], and the other is neutral (dispersal-related) process [19–21]. Compared with neutral processes, previous studies mostly found a significant relationship between bacterioplankton composition and environmental parameters. For instance, Glaeser et al. [22] revealed that singlet oxygen exposure, mediated by dissolved organic matter photolysis, is regarded as an important natural selective factor affecting bacterioplankton structure in aquatic ecosystems. Agogué et al. [23] found that deep-water masses could also influence the bacterioplankton composition in the deep waters of the Atlantic. In fact, at local or regional scales, neutral processes also can explain the bacterial community composition in diverse aquatic environments, but this has seldom been evaluated [7, 24, 25]. Therefore, for a better understanding of the mechanisms that play the predominant ecological role in shaping microbial communities [26–28], besides considering environmental selection, there is also an urgent need to explore the influence of neutral processes on microbial community assembly.

A few studies have compared the relative influences of selective and neutral processes for assembly of abundant and rare microbial subcommunities in different ecosystems [16, 29, 30]. For both abundant and rare bacterial taxa, some studies have suggested that selective processes strongly influence their distribution, whereas neutral processes do not [31]. In contrast, others have argued that the rare bacterial subcommunity is more likely to be affected by local environmental conditions, while abundant taxa are mostly governed by spatial factors (dispersal-related) [7]. Moreover, it has been suggested that rare and abundant microbial taxa possibly have different ecological responses to environmental changes in marine systems [6, 32].

Coastal bays are crucial for urban and industrial development because they have a remarkable influence on both the environment and the economy [33]. Despite the well-recognized ecological importance of these coastal ecosystems, only a few studies have been carried out so far on bacterial biogeography [34], and the relative contributions of selective and neutral processes on the assembly of abundant and rare bacterioplankton subcommunities have not been reported for subtropical bays of China at mesoscales (tens to thousands of kilometers).

In this study, we first hypothesize that the similar biogeographic pattern between abundant and rare bacterioplankton occur in subtropical bay ecosystems. Our second hypothesis is that the neutral processes explain more of the differences in abundant and rare subcommunities assembly along three subtropical bays than does environmental selection. To test these hypotheses, we used high-throughput sequencing technologies to compare the composition and diversity of bacterioplankton community in three subtropical bays (Shenhu Bay, Dongshan Bay, and Beibu Gulf) from southern China.

## Materials and methods

### Study area and sampling

**Fig. 1:**
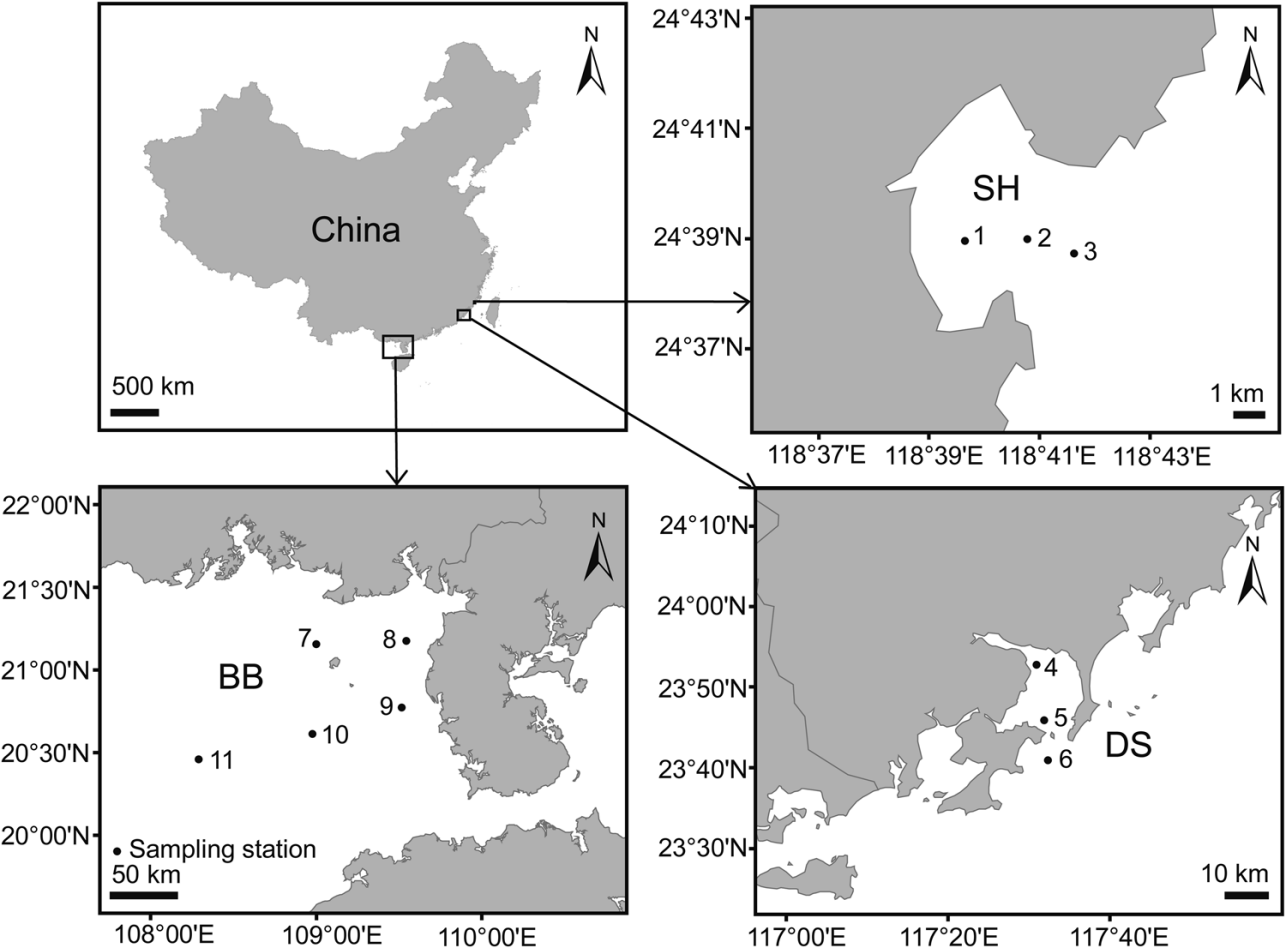
Location of the 11 sampling stations in the three subtropical bays of China investigated in this study. Water samples were simultaneously collected from both surface and bottom layers. SH Shenhu Bay, DS Dongshan Bay, BB Beibu Gulf

Both surface (0.5 m) and bottom (3–45 m) water samples were collected from Shenhu Bay (stations 1, 2, and 3) in May 2012, Dongshan Bay (stations 4, 5, and 6) in August 2011 and Beibu Gulf (stations 7–11) in August 2011 (Supplementary Table S1). The three bays sampled span from 20 to 25 °N (Fig. 1). For bacterioplankton community analyses, 800 ml seawater samples were pre-filtered by a 200-μm sieve to remove debris, macro- and meso-zooplankton, then filtered through a 0.22-μm-pore polycarbonate membrane (47 mm diameter, Millipore, Billerica, MA, USA) following Yu et al. [35]. All membranes were immediately frozen at −20 °C in the field and stored at −80 °C in the lab finally until DNA extraction.

### Physico-chemical analysis

Water temperature, salinity, and depth were measured in situ with conductivity–temperature–depth (CTD) oceanic profilers (SBE-917). Other chemical parameters, including pH, dissolved oxygen (DO), chemical oxygen demand (COD), total nitrogen (TN) (including inorganic and organic nitrogen), nitrite nitrogen (NO_2_-N), nitrate nitrogen (NO_3_-N), ammonium nitrogen (NH_4_-N), soluble reactive phosphorus (SRP, mainly phosphate), total phosphorus (TP, including inorganic and organic phosphorus) and active silicon (DSi) were measured according to the standard methods defined in the Offshore Marine Chemical Survey Technical Regulations [36]. All physico-chemical parameters are given in Supplementary Table S1.

### DNA extraction, PCR, and high-throughput sequencing

Total genomic DNA extraction was performed directly from each membrane using the FastDNA spin kit (BIO101 systems, MP Biomedicals, Solon, OH, USA) following the manufacturer’s instructions. A set of primers were used to amplify the hypervariable V4 region (∼207 bp) of the bacterial 16S ribosomal RNA (rRNA) gene. Each DNA sample was amplified using the primers 16S-F (5′-AYTGGGYDTAAAGNG-3′) and 16S-R (5′-TACNVGGGTATCTAATCC-3′) [35, 37]. The forward and reverse primers were tagged with adapter, pad, and linker sequences. A barcode sequence was added to the reverse primer for pooling of multiple samples in one run of MiSep sequencing [38]. Then triplicate PCR products for each sample were pooled in equal quantity and purified using GeneJET Gel Extraction Kit (Thermo scientific, Hudson, NH, USA) according to the manufacturer’s instructions. Libraries were constructed with NEB Next Ultra DNA Library Prep Kit for Illumina (New England Biolabs, Beverly, MA, USA) following the manufacturer’s recommendations, and index codes were added. The library quality was assessed in Agilent Bioanalyzer 2100 system (Agilent Technologies, Palo Alto, CA, USA). The library was sequenced on an Illumina MiSeq platform (Illumina, Inc., San Diego, CA, USA) using a paired-end approach.

### Taxonomy and OTUs classification

Sequenced paired-end reads were assembled with FLASH [39]. Raw data were processed and analyzed using MOTHUR v.1.33.3 to remove primers and low-quality reads [40]. Sequences were quality controlled using the following settings: we eliminated (1) any sequences length <150 or >300; (2) sequences average quality <30; (3) sequences that contained ambiguous bases >0; (4) homopolymer length >6 [7]. The remaining sequences were aligned to a reference alignment, and those that did not align to the correct region were eliminated, then chimeras were removed using Black Box Chimera Check software (B2C2) [41]. After that, sequences were clustered into operational taxonomic units (OTUs) based on 97% sequence similarity. Representative sequences in each OTU were selected and blasted against the Ribosomal Database Project (RDP) 16S rRNA gene training set (version 9, http://rdp.cme.msu.edu) [42] using the Bayesian classifier at 80% confidence level (if sequence similarity to a reference sequence is <80%, they will be binned to unknown sequences) [43]. Then, these unknown sequences were further analyzed BLAST against NCBI GenBank database (www.ncbi.nlm.nih.gov/blast). Afterward, all chloroplasts, Archaea, Eukaryota and unknown sequences, and singleton OTUs were discarded before the downstream analyses. Eventually, to correct possible errors induced by unequal sequencing efforts in all samples, the OTU table was randomly subsampled to ensure an equal number of sequences per sample (28,923) based on MOTHUR v.1.33.3 [40].

### Definition of abundant and rare taxa

The abundant or rare OTUs were defined following recent publications that combined their local and regional relative abundances [7, 44]. Briefly, locally abundant OTUs were defined as the OTUs with a representation of ≥1% within a sample, whereas locally rare OTUs were defined as having an abundance of <0.01% within a sample. Afterward, regional relative abundances for specific OTUs were calculated as the average of local relative abundances for such OTUs across all samples, including zero values. The thresholds for defining abundant and rare at the regional level were arbitrarily defined as the local thresholds divided by a factor of 10. The OTUs that had a mean relative abundance of ≥0.1% in all samples were defined as regionally abundant OTUs, whereas the OTUs with a mean relative abundance of <0.001% in all samples defined as regionally rare OTUs. Finally, the downstream analyses were performed at three levels: all OTUs, abundant, and rare OTUs.

### Community diversity and structure

Observed richness, abundance-based coverage estimator (ACE), Chao 1, diversity indices (Shannon-Wiener, Simpson index of diversity, and Pielou’s evenness) and rarefaction curves based on the identified OTUs were estimated in R with the vegan package. Good’s coverage was calculated in MOTHUR v.1.33.3 software [40]. Mann–Whitney *U* test was used to compare the difference in OTU relative abundance between abundant and rare subcommunities.

Non-metric multidimensional scaling (NMDS) ordination was performed to explore differences in bacterioplankton communities between bays or stations [45]. An analysis of similarity (ANOSIM) was used to test significant difference in bacterioplankton communities with PRIMER 7.0 [45]. The global R in ANOSIM ranges from 0 to 1 and represents separation degree between groups; *R* = 0 indicates no separation, whereas *R* = 1 suggests complete separation [45].

### Environmental and spatial components

To assess the impacts of environmental filtering, all physico-chemical parameters except pH were log (*x* + 1) transformed to improve homoscedasticity and normality for multivariate statistical analyses [46]. The relationships between the Bray–Curtis dissimilarity of bacterial community (square-root transformed abundances of OTUs) and geographic distance and Euclidean distance of environmental variability were analyzed based on Spearman’s rank correlations. For the spatial component, we followed the approach of the principal coordinates of neighbor matrices (PCNMs) analysis [47, 48] to calculate a set of spatial variables based on the longitude and latitude coordinates of each sampling station.

### Relationships between bacterial communities and environmental and spatial variables

A redundancy analysis (RDA) or canonical correspondence analysis (CCA) was performed to investigate the relationships between bacterial communities and environmental/spatial factors [46], based on the longest gradient lengths of detrended correspondence analysis (DCA). The longest gradient lengths were <3 for all and abundant taxa, indicating RDA is suitable for both communities; while the longest gradient length was >4 for rare taxa, indicating CCA is suitable for rare subcommunity.  Before the RDA or CCA analysis, the environmental variables and PCNMs with high variance inflation factor (VIF) >20 were eliminated to avoid collinearity among factors, and a forward selection was conducted to select those explanatory variables with significant explaining factors (*P* < 0.05) for further analyses [49].

Then, variation partitioning analysis (VPA) was used to evaluate the relative contribution of the selective and neutral processes in shaping marine bacterioplankton community with adjusted *R*^2^ coefficients based on RDA or CCA [50]. The relative contribution of both components was explained by pure spatial variables (*S*|*E*), pure environmental variables (*E*|*S*), spatial variables (*S*), environmental variables (*E*) and the combined effects of both space and environment (*S* ∩ *E*), respectively. In this analysis, the residual proportion represents the unexplained variance.

In addition, Mantel and partial Mantel tests were conducted for relationships between bacterial community dissimilarity and spatial/environmental variables [51]. Although VPA is widely used in ecological research to determine the relative importance of deterministic (selective) versus stochastic (neutral) processes for community structure, several studies based on simulation models showed that VPA failed to correctly predict the environmental and spatial components of community variation [52–54]. Therefore, only VPA could be difficult to use for inferring ecological processes [55], and great caution is needed when using VPA to partition community variation, and it should be used as an exploratory tool together with other approaches (e.g., Mantel test and partial Mantel test) to test hypotheses and assess the relative importance of environmental variables and spatial variables. All statistics analyses were performed in R (http://www.r-project.org) [56].

### Neutral community model

We used Sloan’s community model to assess the potential importance of neutral processes for the entire bacterial community [57], which refers to the OTUs detection frequency in a set of local communities, and their regional relative abundance across the wider metacommunity. This neutral model can reflect adaptation of the neutral theory [20] adjusted to be appropriate for large microbial populations. The parameter *R*^2^ predicts the overall fit to the neutral model. The parameter *Nm* determines the relationship between occurrence frequency and regional relative abundance, with *N* being the metacommunity size and *m* describing immigration rate [57].

### Accession number

The 16S rRNA gene sequences from this study have been deposited in the public NCBI database (http://www.ncbi.nlm.nih.gov/) under the accession number SRP109151 (BioProject accession number PRJNA380540, BioSample accession numbers SAMN07331062– SAMN07331083).

## Results

### Diversity of the bacterioplankton community

We recovered 931,901 high-quality sequences, which clustered into 64,489 OTUs at 97% sequence similarity level. After subsampling to 28,923 sequences per sample, 636,306 sequences (19,975 OTUs) were retained (Table 1). The number of OTUs estimated by Chao1 (20,218 ± 19) and ACE (20,845 ± 56) diversity indices was roughly equivalent to the total number of observed OTUs (19,975) (Table 1). Good’s coverage ranged from 91.15 to 95.65% in each sample and the coverage index of all 22 samples combined was 99.79% (Supplementary Table S2). Although individual samples and those from the three bays did not show a full rarefaction saturation, the rarefaction curve of the pooled data (22 samples) was more saturated, suggesting that we had good coverage of the bacterial richness at global level. The estimated species accumulation curves, extrapolated species richness indices (Chao 1 and ACE), and Good’s coverage indices all support the idea that the great majority of bacterioplankton taxa in the bays were recovered in our samples (Supplementary Fig. S1, Table 1, and Supplementary Table S2).

**Table 1:** General description of all, abundant, and rare OTUs datasets at 97% sequence similarity level

	OTU number	Sequence number	Chao 1	ACE
All OTUs	19,975	636,306	20,218 ± 19	20,845 ± 56
Abundant OTUs	88 (0.44%)	374,126 (58.80%)		
Rare OTUs	15,622 (78.21%)	41,522 (6.53%)		

Abundant OTUs were defined as the OTUs with an abundance ≥1% in a sample (local community) and a mean relative abundance of ≥0.1% in all samples (metacommunity)

Rare OTUs were defined as the OTUs with an abundance <0.01% in a sample and a mean relative abundance of <0.001% in all samples

In total, 88 (0.44%) OTUs with 374,126 sequences (58.80%) were considered to be abundant, while 15,622 (78.21%) OTUs with 41,522 (6.53%) sequences were classified as the rare taxa (Table 1). Only one OTU (25,715 sequences) was always locally abundant (≥1% in all samples), while 14,674 OTUs (37,162 sequences) were always locally rare (<0.01% in all samples) (Supplementary Fig. S2). The richness (OTU number) of rare taxa was two orders of magnitude greater than that of abundant taxa, but the abundances of rare OTUs were about one-tenth of the abundant ones (Table 1).

Proteobacteria was the most diverse bacterial phylum across all samples (27.2% of OTUs), although 33.5% of OTUs could not be classified (Supplementary Fig. S3A). Bacteroidetes taxa were dominant in terms of relative abundance of sequences, along with Alphaproteobacteria and Gammaproteobacteria, because both their richness and abundances were much higher than other bacteria (Supplementary Fig. S3A). In addition, the most diverse and abundant groups in both abundant and rare subcommunities were assigned to Actinobacteria, Bacteroidetes, Cyanobacteria, Planctomycetes, Alphaproteobacteria, and Gammaproteobacteria (Supplementary Fig. S3B). Among these, Bacteroidetes (mean relative abundance: 27.1% OTUs and 30.0% sequences for abundant taxa vs. 18.3% OTUs and 18.5% sequences for rare taxa, respectively), Alphaproteobacteria (16.2% OTUs and 18.7% sequences for abundant taxa vs. 11.8% OTUs and 11.7% sequences for rare taxa, respectively) and Gammaproteobacteria (14.9% OTUs and 13.9% sequences for abundant taxa vs. 10.3% OTUs and 10.3% sequences for rare taxa, respectively) were also the most dominant as entire community (Supplementary Fig. S3B). Yet, these three groups exhibited significantly higher diversity (OTU number) and abundance (sequence number) in the abundant bacteria compared with the rare subcommunity (Supplementary Fig. S3B).

### Geographic patterns of bacterioplankton community

**Fig. 2:**
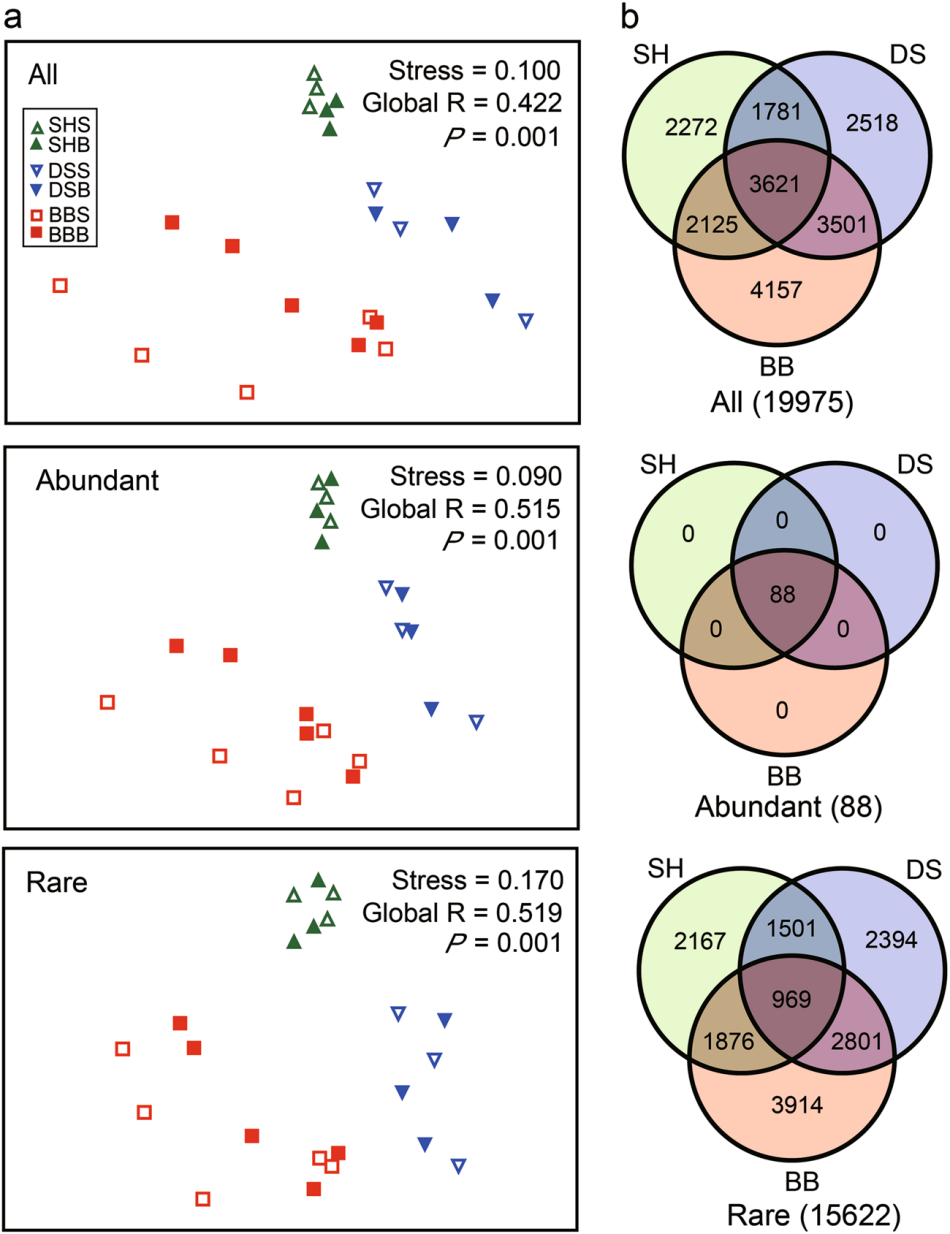
Comparison of bacterioplankton communities among the three subtropical bays of China. **a** Non-metric multidimensional scaling (NMDS) ordination based on Bray–Curtis similarity. Samples in the three regions with two layers (surface and bottom). **b** Venn diagram showing the number of OTUs (19,975 all OTUs, 88 abundant OTUs, 15,622 rare OTUs) that are unique and shared between three subtropical bays. SH Shenhu Bay, DS Dongshan Bay, BB Beibu Gulf, All all bacterial taxa, Abundant abundant taxa, Rare rare taxa

We observed a striking separation of communities for the three studied bays, with their distribution distributed following a clear trend from Shenhu Bay to Dongshan Bay and Beibu Gulf (Fig. 2a). However, we found that the degree of segregation in vertical stratification was relatively low between surface and bottom communities (Fig. 2a). All abundant OTUs (100%) were shared in the three bays, but the percentage of shared OTUs (6.20%) was much lower for the rare taxa (Fig. 2b).

To further explore bacterioplankton distribution, we calculated rank abundance of the 100 most abundant OTUs in each bay. We observed that many of the abundant OTUs in the Shenhu Bay and Dongshan Bay communities were common to both bays (Supplementary Fig. S4A), most of the abundant OTUs in the Shenhu Bay were present in the Beibu Gulf (Supplementary Fig. S4B), and the OTUs that were abundant in the Dongshan Bay were also often abundant in the Beibu Gulf (Supplementary Fig. S4C).

**Fig. 3:**
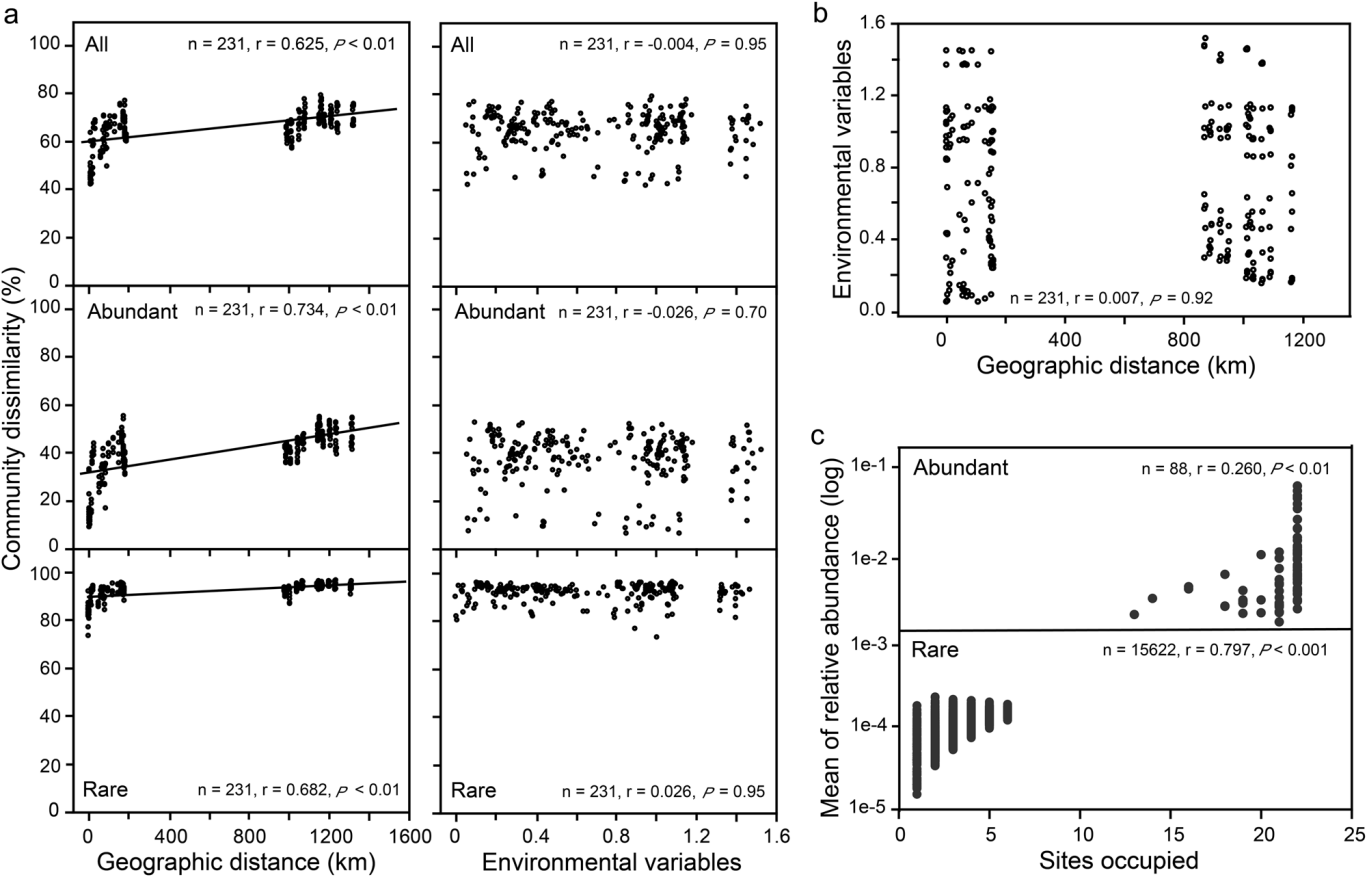
Relationships between bacterioplankton community, geographic distance, and environmental factors. **a** Spearman’s rank correlations between the Bray–Curtis dissimilarity of bacterioplankton community and geographic distance and the Euclidean distance of environmental variables. The *n* is the number of comparison, and all 13 environmental variables were used. **b** Spearman’s rank correlation between the Euclidean distance of environmental variables and geographic distance. **c** Abundance-occupancy relationship of bacterial taxa. Spearman’s rank correlation between mean relative abundance of abundant and rare bacteria and number of samples occupied (*n* is the number of OTUs)

Our results revealed that the dissimilarity in bacterial community composition between any two bays or sites increased with increasing geographic distance (Fig. 3a). The Spearman’s correlation between the Bray–Curtis community dissimilarity and geographic distance showed significantly positive correlations for the bacterial community with a correlation coefficient of 0.625, 0.734, and 0.682 (*P* < 0.01) for all, abundant and rare communities, respectively (Fig. 3a). However, environmental variables did not exhibit any significant relationship (*r* = 0.007, *P* = 0.92) with the geographic distance (Fig. 3b). In addition, no significant correlation was found between environmental variables and bacterial community dissimilarity (Fig. 3a). In terms of relative abundance, the rare subcommunity exhibited a striking difference compared with all and abundant taxa, as shown by the pairwise Bray–Curtis dissimilarity of bacterial community among three subtropical bays, and the ANOSIM comparisons between bacterial communities in distinct bays (Supplementary Fig. S5).

### Abundance-occupancy relationship

The relative abundance of the abundant bacteria and sites occupied were significantly positively correlated (*r* = 0.260, *P* < 0.01). Likewise, rare bacterial relative abundance and local occupancy were positively correlated (*r* = 0.797, *P* < 0.001) (Fig. 3c). All abundant OTUs occupied >50% of samples, but no rare OTU occupied >50% of samples (Fig. 3c).

### Environmental and spatial factors associated with patterns of bacterioplankton community

To explore the balance between selective and neutral processes in bacterial biogeographic patterns, bacterial community assembly, environmental and spatial variables were further analyzed. Specifically, six environmental variables (DO, salinity, NH_4_-N, NO_2_-N, DSi, TN) and five spatial factors (PCNM nos. 1–4 and no. 6) contributed significantly to explain the community composition of the all bacterial community and the abundant bacterial subcommunity by forward selection (*P* < 0.01; Supplementary Fig. S6). However, four local environmental factors (DO, salinity, NH_4_-N, NO_2_-N) and three spatial variables (PCNM no. 1 and nos. 3–4) exhibited significant effects on the variation of the rare bacterial subcommunity (*P* < 0.01; Supplementary Fig. S6).

**Fig. 4:**
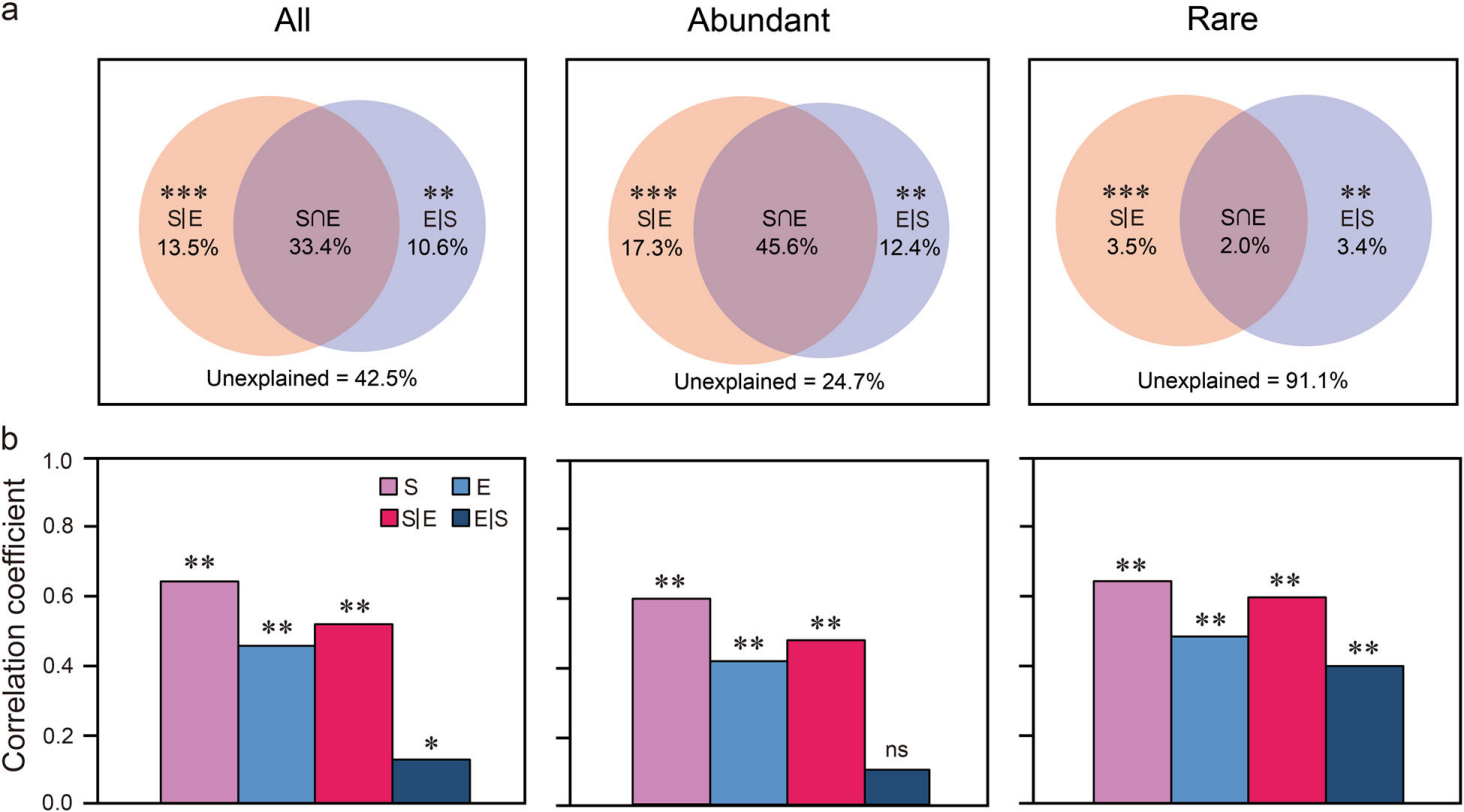
The variation in bacterioplankton metacommunity explained by spatial and environmental variables, respectively. **a** Variation partitioning analysis of the bacterioplankton community composition between spatial and environmental variables. The variation explained by pure spatial and environmental factors corresponds to the bacterial community without the effect of the other by ANOVA permutation tests. **P < 0.01 and ***P < 0.001. **b** Mantel and partial Mantel tests for the correlation between community dissimilarity and environmental and spatial factors using Pearson’s coefficient. *P < 0.05, **P < 0.01. ns not significant, S|E pure spatial variation, E|S pure environmental variation, S ∩ E share explained variation, 1 - S|E - E|S - S ∩ E unexplained variation

VPA revealed that the explained proportion of purely spatial variation (17.3%) in abundant subcommunity composition tended to be higher than purely environmental factors (12.4%). Remarkably, shared environmental and spatial factors explained 45.6% of the variation in the abundant subcommunity, whereas they only explained 2.0% community variation of rare taxa. More importantly, a large amount of the variation (91.1%) in the rare subcommunity was not explained by the spatial and environmental variables, and the relative contribution of purely spatial factors (3.5%) was almost equal to purely environmental parameters (3.4%) (Fig. 4a).

In addition, although both types of factors significantly affected each group of communities by analysis of variance test, spatial variables had much stronger effects on the entire community and the abundant subcommunity compared with rare taxa (Fig. 4a). The Mantel and partial Mantel tests further revealed that spatial factors had stronger influence on bacterioplankton community assembly than environmental factors (Fig. 4b).

## Discussion

### Different diversity but similar biogeographic pattern between abundant and rare bacteria

The diversity differed substantially between the abundant and rare bacterial subcommunities. Campbell et al. [58] reported that Alphaproteobacteria, Gammaproteobacteria, Bacteroidetes, and Actinobacteria were represented in the mostly abundant bacteria from a coastal ocean. Here, we also found that Alphaproteobacteria, Gammaproteobacteria, and Bacteroidetes were dominant, and they showed significant differences between abundant and rare taxa in terms of relative richness and abundance (Supplementary Fig. S3).

So far, studies have reported that the pattern of shared OTUs in abundant microbial subcommunity is complicated among the different regions or sites [31, 44]. For example, our observation was consistent with a study in a mangrove nature reserve that all abundant bacterial taxa (96 OTUs) were shared among three different types of habitats (i.e., cordgrass, ecotone, and mangrove zones) [59]. However, in contrast to our another study that microeukaryotic samples from temperate and subtropical intertidal marine sandy sediments, not all abundant taxa (171 OTUs) were shared among four regions (Connecticut from America, Qingdao, Zhoushan, and Xiamen from China) [16]. Several reasons could account for this phenomenon. First, Alphaproteobacteria, Gammaproteobacteria, Bacteroidetes, Actinobacteria, and Cyanobacteria in our abundant subcommunity were diverse and abundant bacterial groups, as observed from other marine ecosystems [60–62]. The most abundant bacteria can disperse readily as there are, by definition, many more individuals that can potentially be involved in a dispersal event [7]. Interestingly, the frequency distribution of the abundant bacteria showed a significantly positive relationship with the OTUs relative abundance (Fig. 3c), demonstrating that the abundant bacteria increase the probability of dispersal, thereby resulting in a widespread or ubiquitous distribution among the three bays. Second, our result was consistent with Liu et al. [63] but was not in agreement with our another study in marine sandy sediments [16], and the differences may be associated with the size of geographic distance among different study regions, as Liu et al. [59] studied abundant taxa based on small-spatial scale (spanning from 25° 25′ to 25° 29′ N) level like this the present study, whereas our recent study from marine sandy sediments was a large-scale (ranging from 24 to 42 °N) meta-analysis [16]. This means that the smaller the geographic distance is between regions, the higher the similarity of community composition is. So it is reasonable to observe that all abundant bacteria were shared in three subtropical bays.

A total of 6,687 bacterial OTUs could not be classified at sequence similarity >80%. Actually, there were many more unclassified bacteria among the rare OTUs (about 81.8%) compared with abundant taxa (0.2%) in the three subtropical bays. That is, understanding the structure and function of the oceanic microbes has largely focused on taxa that occur in high abundance (abundant taxa) in the past studies, even though most of the marine bacterial species diversity is determined by low abundant or rare taxa [6, 13, 62]. Yet, the highly diverse and rare microbial biosphere remains largely unexplored [6, 7]. For instance, the vast majority of plankton uncovered in samples from the *Tara* Oceans had not previously been identified, with particularly high fractions of novel taxa and genes in the Southern Ocean and in the twilight, mesopelagic zone [62], indicating that the vast majority of the global ocean microbiome still remained to be uncovered [64]. This might partly explain the observed high proportion of unclassified bacterial taxa. However, some of the unclassified bacteria might reflect the potential methodological bias or limitation, such as the short read length of Illumina fragments, so they should be treated with some caution.

Sequencing of over 600,000 V4 rRNA gene sequences from a large dataset (22 samples) was sufficient to approach saturation of bacterial richness, but the number of sequences from each sample is not able to ensure that all OTUs that are present will be always detected (Supplementary Fig. S1). This underscores the effects of sequencing depth. There are always some extremely rare OTUs that cannot be encountered; for example, de Vargas et al. [65] revealed that 1.2 novel metabarcodes were still present in every new 100,000 V9 rDNA reads sequenced (1.2/100,000) from the eukaryotic plankton samples at the global scale.

Recent studies have detected the biogeography of abundant and rare microbial subcommunities in aquatic systems [29, 31, 32, 44]. In this study, bacterial assemblages (all, abundant and rare communities) clearly separated the three subtropical bays according to our NMDS result. Notably, the biogeographic pattern of abundant taxa was similar to that of rare ones (Fig. 2a), although biodiversity pattern largely differed between them (Fig. 2b). The similar biogeographic patterns suggest that environmental changes might be responsible for rare taxa in a similar way as abundant ones [31, 32]. More importantly, bacterioplankton communities from the same region clustered together regardless of the depth where they were sampled, implying that the sequences retrieved from the bottom waters are largely resulting from the surface ocean by sinking or vertical movement of the water mass [14].

Further, both abundant and rare taxa strongly followed distance-decay relationships (Fig. 3a). Yet, the diversity of rare taxa appeared to have more significant differences than abundant ones among the three bays (Supplementary Fig. S5). This is indicative of stronger response to dispersal limitations in rare taxa than in abundant bacterioplankton subcommunities, which could be explained by surface currents or water masses. However, our results are in contrast to Wu et al. [3], who found that abundant taxa had greater dispersal limitations compared with rare taxa in the surface layer of northwestern Pacific Ocean. The disagreement might be attributed to our more narrow area covered in the study and effects of different currents and water masses. This was in accordance with a previous study that found small sample sizes affected metacommunity dynamics [66]. In addition, Liu et al. [7] suggested that lakes and reservoirs that were closer to each other tended to have more similar environmental conditions, but this contrasts with our finding of no significant increase in environmental variability with geographic distance (Fig. 3b). The disparity suggests that the relationship between environmental conditions and geographic distance might be complex in our studied area. Overall, the rare bacterial assemblages consisting of low-abundance members displayed a different diversity, but had a similar biogeographic pattern compared with abundant taxa at intermediate scale.

### Both environmental selection and neutral processes influenced abundant and rare bacterial subcommunities

The most striking result of this study is the effects of both selective and neutral processes on abundant and rare bacterial assemblages across the three subtropical bays. The mechanisms underlying the community assembly, which generate the complicated biogeographic patterns of bacteria, are still a central issue to microbial ecology [67, 68]. However, most studies of biogeographic patterns in various ecosystems only investigated the influence of environmental and spatial factors on the entire bacterial community composition regardless of abundant and rare taxa [15, 34, 66, 69]. Our study provides deeper understanding into whether the relative importance of local environmental (selective) versus spatial (neutral) processes differ for the abundant and rare bacteria. We found that abundant and rare bacterial subcommunities yielded similar distribution patterns although both communities were significantly related to different local environmental variables and spatial factors (Supplementary Fig. S6). This could be attributed to both major mechanisms—environmental selection and neutral processes [16, 31].

On the one hand, environmental factors such as salinity, DO, NH_4_-N, and NO_2_-N were significantly related to variations in the abundant and rare subcommunities (Supplementary Fig. S6). Previous spatiotemporal analysis has shown that nutrient concentrations (e.g., NH_4_-N and PO_4_-P) strongly affected bacterial composition because they are playing a crucial role for the growth and development of bacteria [70]. Moreover, salinity was also suggested as the major determinant affecting the biogeography of abundant and rare bacterial subcommunities across lake and marine ecosystems [32]. However, unlike the abundant bacteria, TN and DSi did not have significant effects on rare taxa (Supplementary Fig. S6). A similar finding was reported by Liu et al. [7] from lakes and reservoirs. They demonstrated that the dynamics of abundant and rare bacterial taxa were constrained by different environmental variables. Hence, abundant and rare bacterial subcommunities may exhibit distinct ecological niches, and the majority of abundant and rare taxa are more likely to play different roles in the typical subtropical ecosystems. On the other hand, spatial factors (neutral-based processes) are another way of shaping the bacterial community other than environmental selection [69, 71, 72]. Indeed, our neutral model explained the large fraction (*R*^2^ = 57%) of the variability in occurrence frequency of entire bacterial community, indicating that neutral processes have a strong role for entire bacterial community assembly (Supplementary Fig. S7). Our results also revealed that spatial variables significantly affected abundant and rare subcommunities structure in line with significant distance-decay relationship (Fig. 3a and Supplementary Fig. S6). Galand et al. [13] surveyed rare microbial biosphere of the Arctic Ocean and showed rare phylotypes followed patterns similar to those of the most abundant members of the community and of the entire community. This observation was consistent with our results. Interestingly, this finding was also similar to recent conclusions from bacterioplankton communities in a unique system of coastal Antarctic lakes [32], bacterioplankton of the lakes and reservoirs from China [7], and bacteria in lakes on Yungui Plateau of China [31], respectively.

Although environmental selection and neutral processes were critical to both abundant and rare taxa, both types of drivers and their individual contributions significantly differed (Fig. 4a, b). We found that purely environmental components were slightly less influential contributors than the purely spatial variables in regulating the assembly of both abundant and rare subcommunities, although the community variation that was purely explained by environmental or spatial variables was significant (Fig. 4a). In addition, partial Mantel tests also corroborated that pure spatial factors more strongly explained both subcommunities than pure environmental variability (Fig. 4b). Our result was consistent with a recent study on marine abundant and rare picoeukaryotic subcommunities in 40–75 m depth subsurface layers from northwestern Pacific Ocean [3]. However, in contrasted to that from Liao et al. [31], which suggested that both abundant and rare bacterial taxa were strongly influenced by deterministic process (environmental selection) but were weakly influenced by neutral processes. These differences possibly resulted from different environmental gradients and geographic scale differences among the studied areas. For example, Logares et al. [32] suggested environmental filtering (e.g., salinity) strongly drove abundant and rare bacterial subcommunities composition but geographic distance played a weak role on both taxa between lakes. This contrasting result is likely due to a large salinity gradient (0–100 psu) very different to the gradient in our study (30.57–35.14 psu), implying that large environmental gradients can affect the assembly of abundant and rare bacterial subcommunities. In addition, Zhang et al. [16] found that both abundant and rare microeukaryotic subcommunities showed a slightly stronger response to environmental factors than to spatial (distance) factors. This might be because they studied the biogeography of communities along a large spatial scale (latitudinal gradient ranging from 24 to 42 °N) rather than at intermediate scale covered in the present study (latitudinal spanning from 20 to 25 °N), suggesting different spatial scales provide another way to generate differences in abundant and rare subcommunities assembly mechanisms.

More interestingly, the spatial effect on the variation of abundant taxa was stronger than that on its rare counterparts (Fig. 4a). This observation was in agreement with a previous study on aquatic and sedimentary microbial communities in the lakes of western China [30]. In fact, the discrepancy from both taxa may be explained from two aspects. First, the abundant taxa with higher dispersal rates are intensified by drift (neutral processes) or priority effects [73], and the generalists present in the abundant subcommunity [31]. Second, the variation of rare bacteria purely explained by both the spatial and the environmental factors is relatively low because about 91.1% of the variation is unexplained (Fig. 4a). The large ratio of unexplained variation could be attributed to other biotic interactions such as competition and trophic interactions [74]; another plausible explanation is that unmeasured environmental and biological factors or methodological issues [75]. For instance, tides, upwelling and surface winds and the movement of currents and water masses [76], which were not measured in this study. Clearly, these findings highlight that integrating more environmental factors and extending the sampling sites, even performing manipulative experiments across space and time, are crucial for further understanding the mechanisms that mediate the balance between deterministic (environmental selection) and neutral processes on the biogeography of abundant and rare bacterioplankton.

## Conclusions

Our study provides important insights for explaining microbial community patterns in subtropical marine ecosystems for both abundant and rare taxa. Different diversity but similar biogeographic patterns were detected between abundant and rare taxa in three subtropical bays of China. More importantly, both selective and neutral processes seemed to drive the abundant and rare subcommunities assembly, although their relative contributions on both subcommunities composition were different. However, spatial factors explained more community variation in abundant bacteria than that in rare bacteria. Altogether, this study highlights the importance of considering environmental selection and neutral processes to understand abundant and rare subcommunities assembly and biogeography. The conclusion is, in contrast to other studies, that different ecological driving forces govern the biogeography of abundant and rare bacterial subcommunities, implying more complex mechanisms may be at play to shape both subcommunities, and tremendous diversity of the rare biosphere may be subjected to more complicated ecological processes such as speciation, drift, and extinction. Therefore, further experiments are necessary to distinguish and disentangle potential assembly mechanisms between the two groups across various space and time scales.

## Electronic supplementary material

Supplementary Information
