# Supplementary material for: Biogeographic patterns of abundant and rare bacterioplankton in three subtropical bays resulting from selective and neutral processes
Source: ISME J. 2018 Jun 7;12(9):2198–210. doi: 10.1038/s41396-018-0153-6 (PMC6092436; doi:10.1038/s41396-018-0153-6)
Supplement: Supplementary file 1 — Supplementary Information [file 41396_2018_153_MOESM1_ESM.pdf]

Supplementary Information

Biogeographic patterns of abundant and rare bacterioplankton in three subtropical bays resulting from selective and neutral processes

Yuanyuan Mo[1], Wenjing Zhang[1], Jun Yang[2], Yuanshao Lin[1], Zheng Yu[2,3], Senjie Lin[1,4]

[1]State Key Laboratory of Marine Environmental Science, Marine Biodiversity and Global Change Research Center, College of Ocean and Earth Sciences, Xiamen University, Xiamen 361102, China
[2]Aquatic EcoHealth Group, Key Laboratory of Urban Environment and Health, Institute of Urban Environment, Chinese Academy of Sciences, Xiamen 361021, China
[3]Department of Chemical Engineering, University of Washington, Seattle, WA, USA
[4]Department of Marine Sciences, University of Connecticut, Groton, CT, USA

Correspondence authors

Wenjing Zhang. **E-mail**: Zhangwenjing@xmu.edu.cn;
Jun Yang. **E-mail**: jyang@iue.ac.cn

The supplementary information includes:

- 10 pages
- 7 figures
- 2 tables

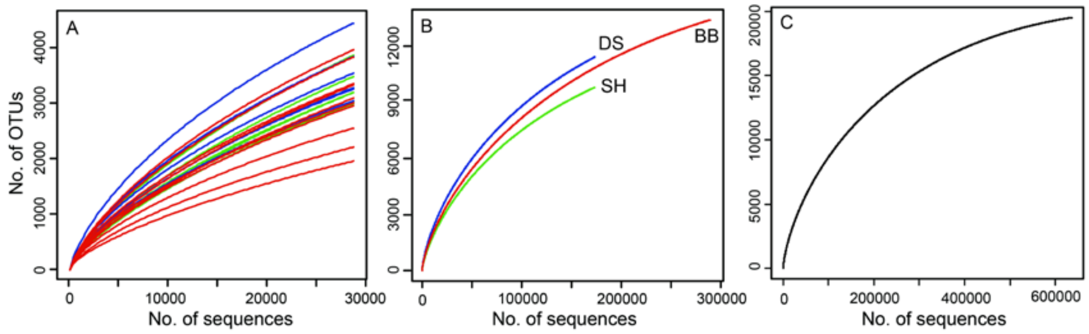

Fig. S1 Rarefaction curves of similarity-based operational taxonomic units (OTUs) at 97% similarity level. (A) The individual samples (local communities), (B) the combined sets of each of three bays samples (6 samples in both SH and DS, and 10 samples in BB), (C) the combined set of 22 samples (metacommunity). SH, Shenhu Bay; DS, Dongshan Bay; BB, Beibu Gulf.

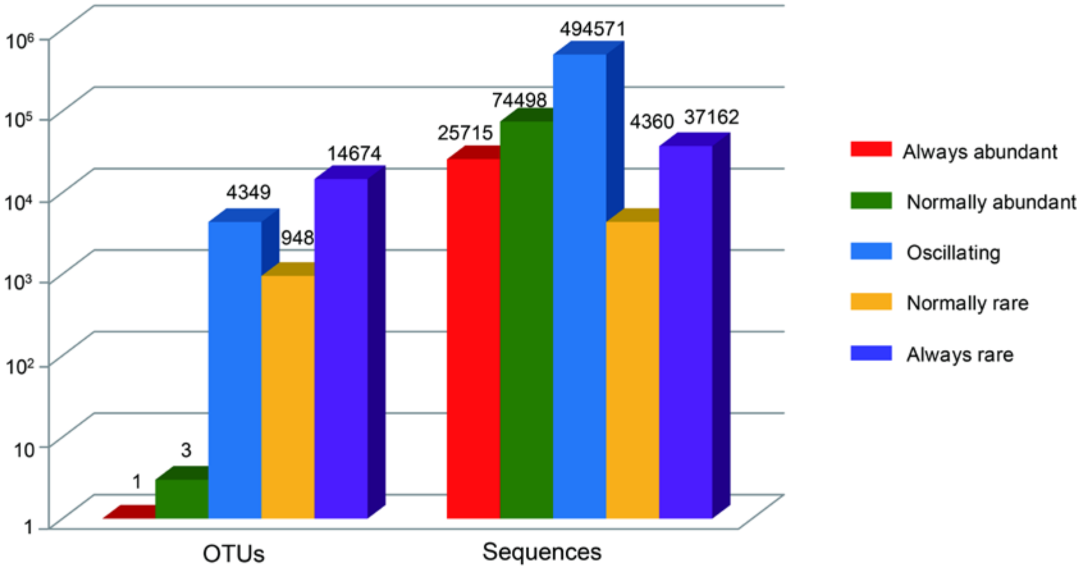

Fig. S2 The number of OTUs and sequences of always abundant (the OTUs with ≥ 1% abundance in all samples), normally abundant (the OTUs with ≥ 1% abundance were present ≥ 70% of the samples), Oscillating (the OTUs does not fall in any of the other categories), normally rare (the OTUs with < 0.01% abundance were present ≥ 70% of the samples), and always rare bacteria (the OTUs with < 0.01% abundance in all samples).

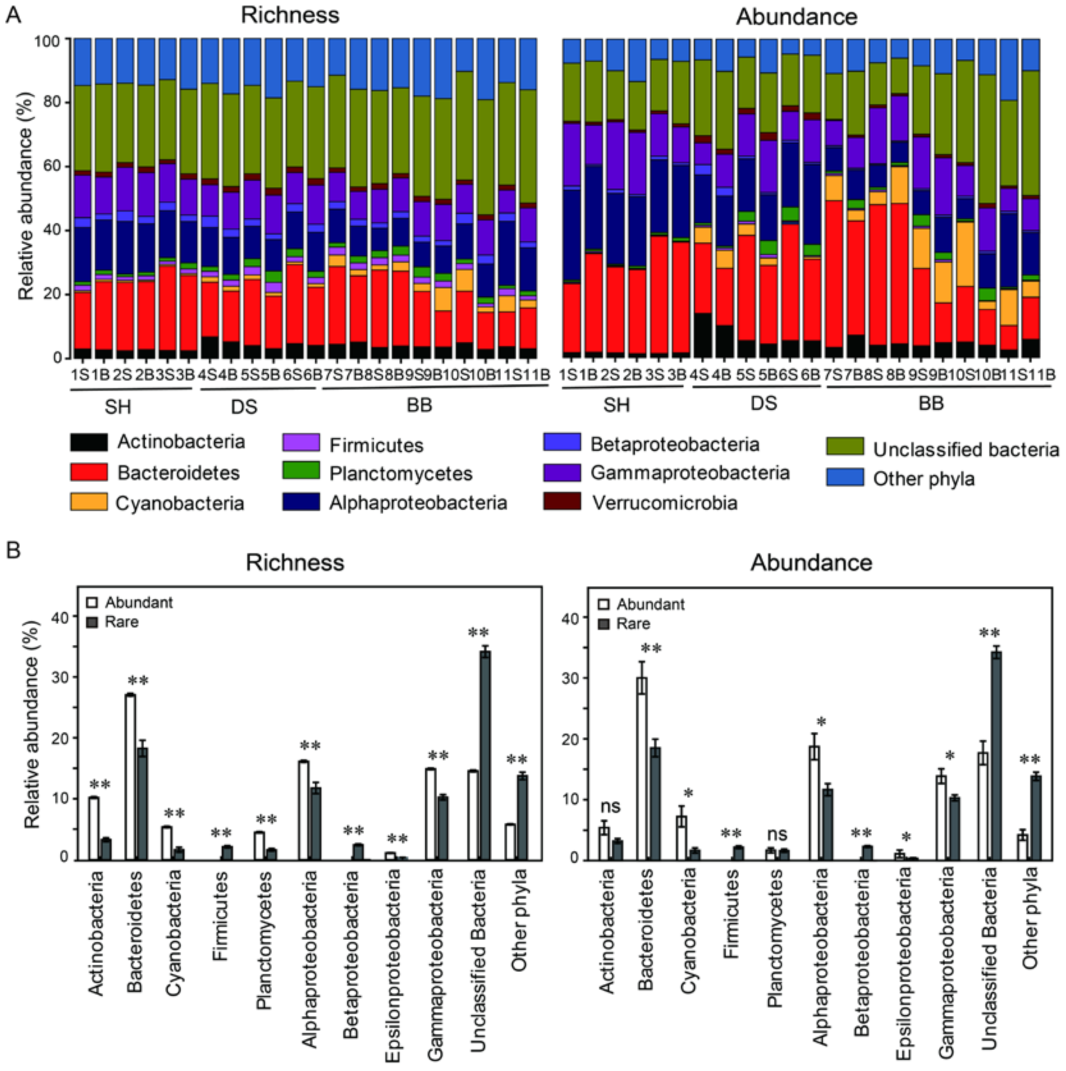

Fig. S3 The known and unknown components of bacterioplankton community biodiversity and composition. (A) Richness (OTU number) and abundance (sequence number) of major bacterioplankton taxonomic groups across 22 samples. SH, Shenhu Bay; DS, Dongshan Bay; BB, Beibu Gulf. (B) Relative abundance of richness (OTU number) and abundance (sequence number) of abundant bacterial taxa compared with rare taxa in three subtropical bays of China. Values and error bars indicate means and standard errors, respectively. ns - not significant, **P* < 0.05, ***P* < 0.01 (Mann-Whitney *U* test).

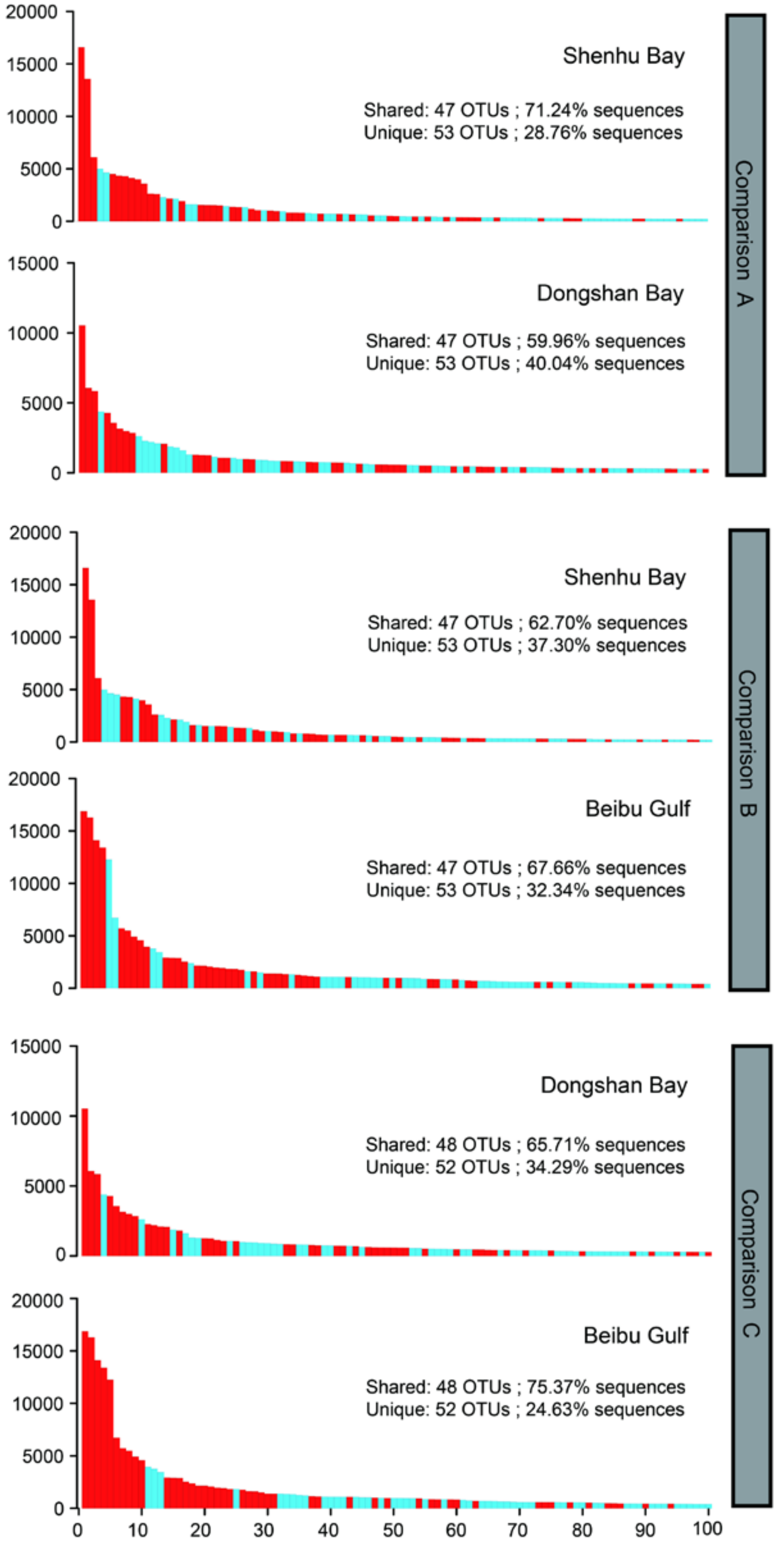

Fig. S4 Rank abundance histograms of bacterioplankton community overlap between (A) Shenhu Bay and Dongshan Bay, (B) Shenhu Bay and Beibu Gulf, and (C) Dongshan Bay and Beibu Gulf. OTUs highlighted in red are shared between two compared regions, whereas OTUs highlighted in blue are unique to each region.

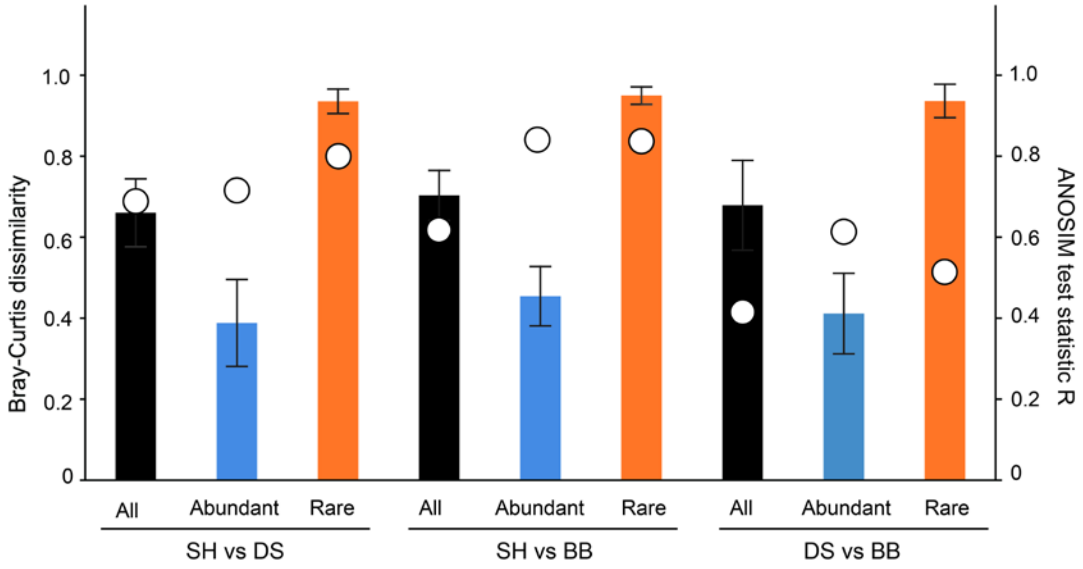

Fig. S5 ANOSIM and community dissimilarity for the all, abundant and rare bacterioplankton between three subtropical bays. Given is the ANOSIM test statistic (R, denoted as circles) that two compared groups are significantly different at the $P < 0.05$ level (all tests were significant). ANOSIM *R*- and *P*-values were generated using the Bray-Curtis dissimilarity. SH, Shenhu Bay; DS, Dongshan Bay; BB, Beibu Gulf. Data are means±s.d. (error bars).

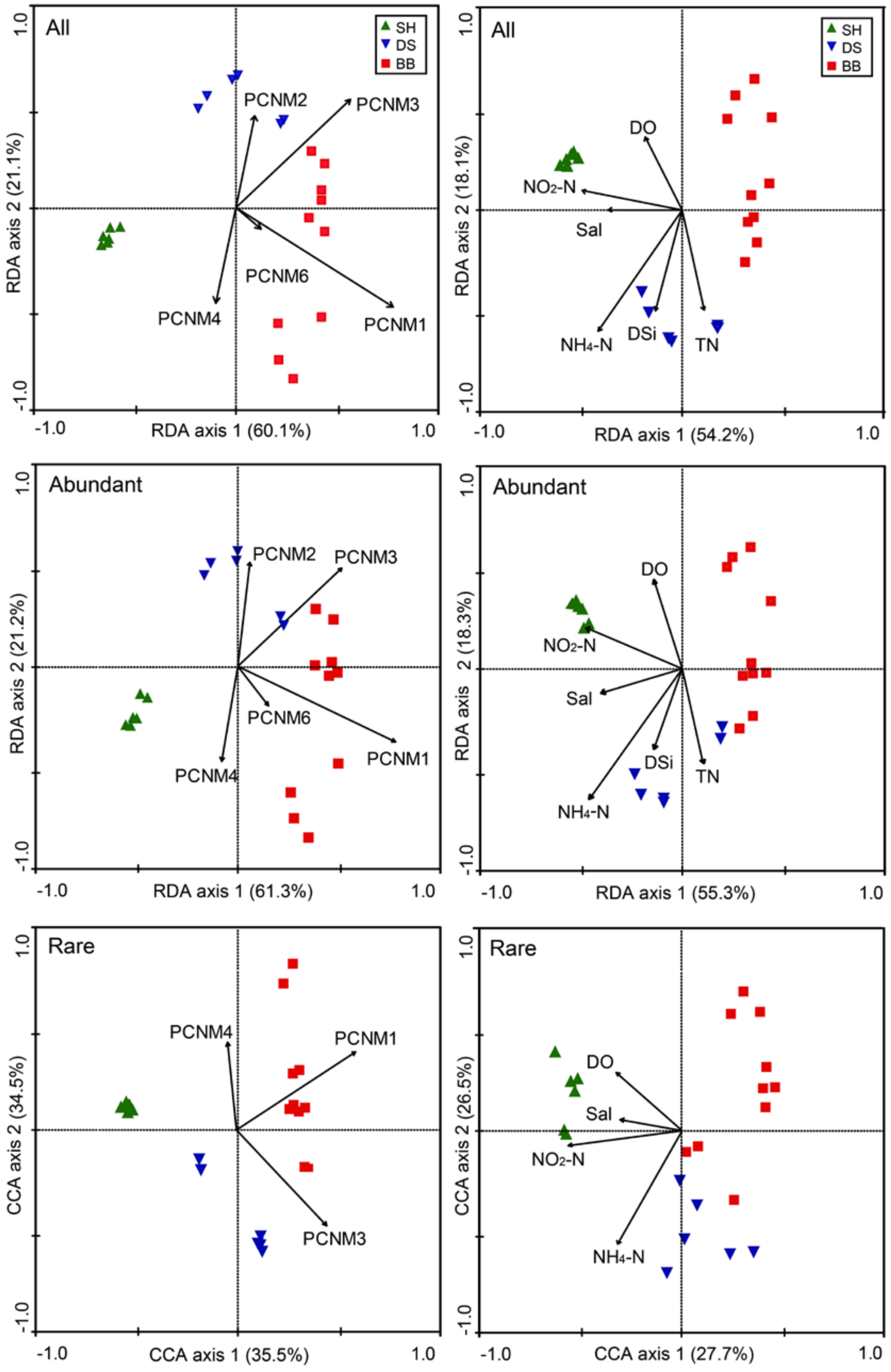

Fig. S6 CCA/RDA ordinations showing the bacterioplankton community composition in relation to significant (P < 0.01) spatial (left-side panels) and environmental variables (right-side panels), respectively.

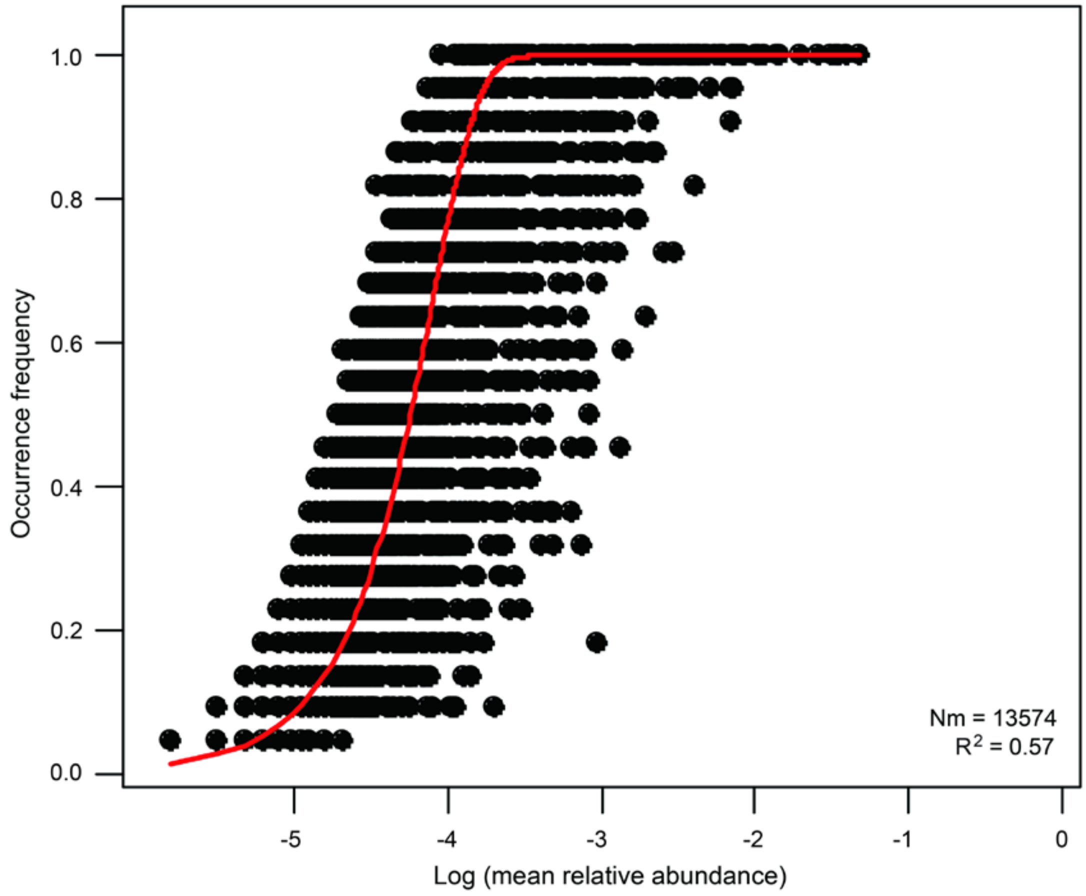

Fig. S7 Fit of the neutral model based on the all bacterioplankton from three subtropical bays. Occurrence frequencies of different OTUs as a function of mean relative abundance in all data sets (*n* =19975). *Nm* indicates metacommunity size times immigration, and R^2 indicates the fit to the neutral model. Note that the X axis was log10 transformed.

Table S1 Environmental variables of 22 samples in the three subtropical bays of China.

Samples / Factors	Shenhu Bay (Summer 2012)						Dongshan Bay (Summer 2011)						Beibu Gulf (Summer 2011)									
	1S	1B	2S	2B	3S	3B	4S	4B	5S	5B	6S	6B	7S	7B	8S	8B	9S	9B	10S	10B	11S	11B
Latitude (°N)	24.65	24.65	24.65	24.65	24.65	24.65	23.88	23.88	23.77	23.77	23.69	23.69	21.17	21.17	21.19	21.19	20.79	20.79	20.63	20.63	20.47	20.47
Longitude (°E)	118.66	118.66	118.68	118.68	118.69	118.69	117.52	117.52	117.53	117.53	117.54	117.54	109.02	109.02	109.56	109.56	109.53	109.53	108.99	108.99	108.31	108.31
pH	8.09	8.10	8.06	8.05	8.08	8.08	8.15	8.17	8.17	8.20	8.23	8.20	8.12	8.08	8.16	8.14	8.07	8.01	8.18	8.03	8.10	8.07
Temperature (°C)	22.40	21.03	22.10	22.00	22.60	21.740	29.20	29.30	26.40	24.00	23.90	22.60	30.84	30.46	30.68	30.68	30.68	30.69	29.98	26.83	29.81	24.86
Depth (m)	0.5	12.0	0.5	13.0	0.5	12.0	0.5	3.0	0.5	9.4	0.5	18.3	0.5	16.0	0.5	11.0	0.5	13.0	0.5	34.0	0.5	41.0
Salinity	32.80	33.12	32.80	33.14	32.90	34.81	30.57	30.80	33.96	34.95	34.97	35.14	31.78	32.56	31.62	31.63	32.36	32.36	33.09	33.23	32.85	33.16
DO (mg/ L)	6.75	6.57	6.69	6.56	6.99	6.78	6.21	6.49	5.40	4.83	5.01	4.73	6.49	5.68	6.44	6.40	5.92	5.95	6.42	4.83	6.44	5.73
COD (mg/ L)	0.340	0.160	0.220	0.400	0.150	0.160	1.050	1.150	0.620	0.870	0.460	0.590	0.198	0.340	0.257	0.250	0.108	0.104	0.149	0.057	0.141	3.822
TN (mg/ L)	0.213	0.169	0.223	0.148	0.200	0.270	0.378	0.414	0.585	0.346	0.208	0.197	0.190	0.206	0.363	0.137	0.120	0.410	0.327	0.101	0.233	0.151
NO_2-N (mg/ L)	0.020	0.020	0.017	0.017	0.016	0.016	0.008	0.008	0.008	0.010	0.008	0.007	0.002	0.008	0.001	0.001	0.021	0.023	0.002	0.014	0.001	0.004
NO_3-N (mg/ L)	0.068	0.068	0.058	0.064	0.057	0.062	0.111	0.106	0.058	0.072	0.045	0.041	—	0.005	0.001	—	0.033	0.024	0.175	0.037	0.002	0.006
NH_4-N (mg/ L)	0.019	0.027	0.032	0.034	0.026	0.033	0.022	0.027	0.063	0.054	0.048	0.051	0.009	0.010	0.036	0.010	0.013	0.014	0.013	0.007	0.007	0.005
TP (mg/ L)	0.028	0.022	0.031	0.084	0.040	0.056	0.078	0.095	0.043	0.089	0.039	0.048	0.022	0.015	0.033	0.048	0.051	0.077	0.026	0.019	0.023	0.029
SRP (mg/ L)	0.007	0.009	0.007	0.007	0.002	0.002	0.006	0.010	0.016	0.022	0.029	0.028	0.001	0.002	0.001	0.001	0.004	0.003	—	0.010	—	0.012
DSi (mg/ L)	0.212	0.160	0.281	0.185	0.391	0.391	1.540	1.280	0.785	0.778	0.720	0.695	0.104	0.105	0.003	0.017	0.049	0.082	0.026	1.118	0.024	0.199

The sample names are indicated using the format: station and layer (surface and bottom). S, surface; B, bottom; "—" indicates that the value < 0.001.

DO, dissolved oxygen; COD, chemical oxygen demand; TN, total nitrogen; NO_2-N, nitrite nitrogen; NO_3-N, nitrate nitrogen; NH_4-N, ammonium nitrogen; TP, total phosphorus; SRP, soluble reactive phosphorus; DSi, active silicon.

Table S2 Diversity of bacterioplankton community from 22 samples of three subtropical bays in China

Sample	Region	Depth (m)	OTU richness	ACE	Chao1	Shannon-Wiener	Simpson index of diversity	Pielou´s evenness	Good's coverage
1S	SH	0.5	3120	8579	7851	5.211	97.59%	64.76%	92.94%
1B	SH	12.0	3997	10352	9031	5.726	98.02%	69.05%	91.27%
2S	SH	0.5	3304	9202	7724	5.244	97.81%	64.72%	92.54%
2B	SH	13.0	3093	8158	6941	5.145	97.38%	64.02%	93.21%
3S	SH	0.5	3602	9452	8086	5.466	97.66%	66.75%	92.09%
3B	SH	12.0	3308	8619	7950	5.346	97.42%	65.96%	92.72%
4S	DS	0.5	3370	7926	6777	5.735	98.89%	70.60%	93.04%
4B	DS	3.0	3972	8800	8011	6.037	98.96%	72.85%	91.95%
5S	DS	0.5	3663	8595	7906	5.795	98.54%	70.62%	92.40%
5B	DS	9.4	4595	9365	8880	6.511	99.31%	77.21%	91.15%
6S	DS	0.5	3394	8213	7328	5.466	97.92%	67.24%	92.82%
6B	DS	18.3	3149	7563	6943	5.290	96.97%	65.68%	93.41%
7S	BB	0.5	2287	5346	4819	4.498	93.57%	58.14%	95.25%
7B	BB	16.0	3061	8056	7321	5.313	97.95%	66.19%	93.33%
8S	BB	0.5	3442	8569	7792	5.114	96.07%	62.80%	92.52%
8B	BB	11.0	3199	8544	7631	5.048	96.80%	62.55%	92.87%
9S	BB	0.5	3964	9419	8612	5.691	98.17%	68.69%	91.67%
9B	BB	13.0	4097	9151	8074	5.882	98.44%	70.71%	91.73%
10S	BB	0.5	2028	5119	4435	4.432	94.75%	58.20%	95.65%
10B	BB	34.0	3112	7747	6668	5.597	98.44%	69.58%	93.47%
11S	BB	0.5	2642	5968	5131	5.267	98.21%	66.85%	94.64%
11B	BB	41.0	3466	8333	7514	5.681	98.23%	69.70%	92.74%
Total	All bays		19975	20218	20845	6.302	98.92%	63.64%	99.79%

SH, Shenhu Bay; DS, Dongshan Bay; BB, Beibu Gulf. The OTU was defined based on 97% sequence similarity level.
